# Interrater Agreement Between Resident‐Self Assessments and Proxy Rated Assessments of Older Persons' Experience of Thriving in Care Homes

**DOI:** 10.1002/nop2.70929

**Published:** 2026-11-13

**Authors:** Ann‐Sofie Silvennoinen, Edvard Johansson, Rebecca Baxter, Harriet Finne‐Soveri, Ådel Bergland, David Edvardsson, Lisbeth Fagerström

**Affiliations:** ^1^ Faculty of Science and Engineering, Caring Sciences Åbo Akademi University Vaasa Finland; ^2^ Faculty of Social Sciences, Business, and Economics Åbo Akademi University Turku Finland; ^3^ Department of Nursing Umeå University Umeå Sweden; ^4^ Finnish Institute for Health and Welfare Helsinki Finland; ^5^ Lovisenberg Diaconal University College Oslo Norway; ^6^ Swinburne University of Technology Melbourne Australia; ^7^ Gothenburg Centre of Person‐Centred Care Gothenburg University Gothenburg Sweden; ^8^ Edith Cowan University Perth Australia

## Abstract

**Aim:**

The aim of this study was to explore the inter‐rater agreement between resident self‐assessments and proxy assessments obtained from relatives and staff concerning thriving in care homes.

**Design:**

A cross‐sectional study.

**Methods:**

The thriving of older persons in care homes from a Finnish city was measured using the short‐form Thriving of Older People Assessment Scale. Descriptive statistics, linear mixed‐effects model analyses, and quadratically weighted Cohen's kappa coefficient calculations were performed.

**Results:**

Interrater agreement levels concerning thriving between residents and staff were generally higher than between residents and relatives or between relatives and staff.

**Conclusion:**

Most residents in care homes for older persons have cognitive impairment. Proxy assessments are needed to make their voices heard. Relatives should perhaps be given a greater role in aged care, and the co‐operation and communication between relatives and staff concerning residents' everyday lives should be improved.

**Implications for the Profession and Patient Care:**

By considering proxy reports, staff and decision‐makers could improve the quality of aged care. Culture change at the organizational level is needed to involve relatives in residents' care to improve thriving.

**Reporting Method:**

The STROBE guidelines.

**Patient and Public Contribution:**

No patient and public contribution.

## 1 Introduction

Long‐term care facilities (LTCF) for older persons offer residents, including those with functional impairments, care that enables a life with basic rights, fundamental freedoms, and human dignity (WHO 2022). Whether care home residents obtain high‐quality care is an important social issue. In nursing science, caring is an essential theoretical concept. Particularly in aged care, compassionate love and mercy are needed now more than ever (Fagerström and Maizels 2019). Older persons should receive individualized care that is adapted to their personal needs and values (Schweighart et al. 2022).

Thriving of older persons living in care homes has been defined as place‐related well‐being that emerges from the interaction between a person and the living environment (Bergland et al. 2014; Bergland and Kirkevold 2001, 2006). Since a high proportion of older persons living in care homes and long‐term care settings experience cognitive impairment (Roitto et al. 2024), careful consideration of assessment methods is required to capture their experience of thriving. Research that involves persons with impaired capacity for decision‐making is ethically challenging (Kim et al. 2011; Usman et al. 2019). Residents with impaired memory may be unable to recall events related to their care, assess its quality, or express their preferences and need for help. The use of proxy information may be necessary to collect data from cognitively impaired care home residents for thriving research as a crucial, albeit second best, source of information (Bergland et al. 2014).

Proxy respondents have been used in scientific studies (Kane et al. 2005; Neumann et al. 2000; Robertson et al. 2019, 2020), and the reliability of proxy information can be assessed by comparing data from the residents and their proxies. According to Howland et al. (2017), the reports of patients and their proxies usually agree when activities of daily living (ADL) are concerned but differ regarding cognitive and executive functions. In a study by Kahanpää et al. (2019), proxies showed a tendency to assess the quality of care as poorer for residents with greater cognitive decline. Assessments made from different perspectives can also contribute to a better overall picture of care quality. However, proxy assessments in care homes can be unreliable and should preferably be performed by proxies who know the person with cognitive impairment well and can provide reliable information. For this reason, proxy reports between staff and relatives may differ systematically (Robertson et al. 2017, 2019). If the relatives seldom visit the resident in the care home, it may be difficult for them to assess care quality and thriving for the resident. The same may be true for staff if their contact with the resident does not have continuity due to working shifts or staff turnover. Agreement with self‐ratings is typically low to moderate and varies by rater (Robertson et al. 2017, 2019; Buchholz et al. 2024). Two approaches to quality of life (QoL) can be distinguished: a care environment‐oriented approach that captures everyday life in context, including relationships and environmental factors (e.g., Robertson et al. 2017, 2019; Rand et al. 2017), and a health‐related approach that measures health using standardized instruments (Pickard and Knight 2005).

In the present paper, we explore levels of interrater agreement between resident self‐assessments and staff and relative proxy assessments of thriving, as measured by the short‐form Thriving of Older People Assessment Scale (TOPAS). To our knowledge, no systematic comparison studies involving resident, relative, and staff assessments of thriving based on the short‐form TOPAS have been performed previously.

## 2 Background

In nursing science, the concept of thriving has received less attention than QoL, which concerns well‐being in general, while thriving focuses on well‐being in relation to the environment (Bergland and Kirkevold 2001, 2006; Bergland et al. 2014, 2015; Björk et al. 2017). In Scandinavian LTCFs, thriving is a commonly used concept in everyday language, referring to a subjective experience of well‐being and contentment, having positive relationships, and settling in (Bergland et al. 2014; Bergland and Kirkevold 2006). In a meta‐ethnography by two main themes and four subthemes on thriving were described: ‘ingredients for thriving’ as a main theme, referring to personal and social contributors as subthemes, and ‘environment for thriving’, referring to spatial and societal contributors. The contributors to thriving appeared to be connected to the resident's lived experience of situated contentment (Baxter, Corneliusson, et al. 2021).

According to Bergland and Kirkevold (2006), older persons have the right not only to survive but also to thrive in care homes. In their study, the residents, having adjusted their expectations to what was possible in the care home, experienced thriving differently in the care home as compared to their previous life. According to Baxter, Corneliusson, et al. (2021), staff could recognize thriving among residents through understanding, observing, and sensing. Staff described that they determined if a resident was thriving or not by putting together information from personal and clinical interactions in relation to the resident and how they interacted with their lived context. The thriving concept may promote planning of care that offers positive experiences of everyday life for residents, and that not only focuses on mitigating medical symptoms and suffering (Bergland et al. 2015).

In the study by Bergland and Kirkevold (2005), appropriate care given by staff members contributed to thriving only for some residents but made life easier for all of them. Silvennoinen et al. (2025) evaluated the thriving of care home residents as correlated to central interRAI scales and demographic characteristics and observed statistically significant, positive associations of thriving with social engagement and female gender. According to Patomella et al. (2016), thriving appears to be associated with care home residents' functional capacity and possibilities to create a varied daily life. In their study, thriving showed positive associations with involvement and higher capacity in ADL and better cognitive functioning. Neuropsychiatric symptoms (NPS) appear negatively associated with thriving, irrespective of cognitive functioning (Baxter et al. 2022; Björk et al. 2018). Knowledge of thriving‐related factors could help staff recognize residents having low levels of thriving and activate all residents, including those with severe cognitive impairment.

Research focusing specifically on thriving of care home residents is scarce, and residents with severe cognitive impairment are an underrepresented population in research (Shiells et al. 2020). For research findings to be useful, there is a need to understand differing proxy views (Robertson et al. 2017). Rater values, relationships, and prior understanding shape proxy assessments and contribute to systematic differences. Staff tend to provide higher ratings, whereas relatives tend to provide lower ratings, potentially reflecting different frames of reference, such as focus on care quality versus comparisons with the resident's previous life (Robertson et al. 2019). Assessments also vary depending on whether a proxy‐patient perspective or a proxy‐proxy perspective is adopted (Engel et al. 2024). Although the resident's perspective is central, a comprehensive understanding of resident thriving requires the inclusion and comparison of both relative and staff assessments.

## 3 The Study

### 3.1 Aim

The aim of this study was to explore the interrater agreement between resident self‐assessments and proxy assessments obtained from relatives and staff concerning thriving in care homes.

### 3.2 Research Questions

1. To what extent is there interrater agreement between resident and staff TOPAS ratings?
2. To what extent is there interrater agreement between resident and relative TOPAS ratings?
3. To what extent is there interrater agreement between relative and staff TOPAS ratings?

## 4 Methods

### 4.1 Design

This study used a cross‐sectional design. The STROBE checklist was used to guide the reporting for this study (Appendix S1).

### 4.2 Instruments With Validity and Reliability

The questionnaires used in this study were the short‐form TOPAS (Bergland et al. 2014; Baxter et al. 2019) and the interRAI‐LTCF version 9.0 (interRAI 2023).

#### 4.2.1 The Thriving of Older People Assessment Scale

The short‐form TOPAS consists of 15 items and has been shown to have sound validity and reliability to measure resident thriving in care homes (Baxter et al. 2019). As in the original 32‐item version of TOPAS, the short‐form TOPAS items are scored on a 6‐point Likert‐type scale ranging from 1 = ‘No, I disagree completely’ to 6 = ‘Yes, I agree completely’ (Baxter et al. 2019; Bergland et al. 2014). Hence, the total short‐form TOPAS score can range between 15 (the lowest possible level of thriving) and 90 (the highest possible level of thriving). The short‐form TOPAS has been validated using staff proxy ratings of resident thriving, showing a high level (*α* = 0.90) of internal consistency (Baxter et al. 2019). The short‐form TOPAS has not yet been validated in Finnish. In this study we therefore applied a forward‐backward translation (Beaton et al. 2000) of the Swedish version to obtain a Finnish version. Given the original validation in Sweden, the cultural proximity, and the fact that approximately half of the participants were Swedish‐speaking, the translated version of the instrument was deemed suitable for assessing resident thriving in Finnish care homes.

#### 4.2.2 The Resident Assessment Instrument

The Resident Assessment Instrument (RAI) produces client information on functional capacity, ability to cope with everyday life, and service needs by using several reliable assessment scales (Hawes et al. 1997; Hirdes et al. 2008). The interRAI‐LTCF is a comprehensive, standardized assessment of residents' needs, strengths, and preferences in long‐term care (interRAI 2023). The central interRAI scales included in this study were Activities of Daily Living Hierarchy (ADL‐H) (interRAI 2023; Morris et al. 1999), Changes in Health, End‐stage disease, and Symptoms and Signs Scale (CHESS) (Hirdes et al. 2014; interRAI 2023), Cognitive Performance Scale (CPS) (interRAI 2023; Morris et al. 1994), Depression Rating Scale (DRS) (Burrows 2000; interRAI 2023), Pain or Pain Scale (Fries et al. 2001; interRAI 2023), and Social Engagement Scale (SES) (interRAI 2023; Mor et al. 1995; Schroll et al. 1997). Data from a statutory RAI assessment, performed during data collection, were used in this study to characterize the participants.

### 4.3 Sampling

All four public care homes for older persons with the municipality as the service provider in a medium‐sized (50,000 inhabitants) Finnish city were invited to participate in this study. The care homes had staff available 24 h a day. The service included nursing care, meals, cleaning, laundry services, and other services such as assistance in‐ and outside the apartment. The services did not include rent, medications, personal hygiene items, clothes, or other personal items (FINLEX 604/2022 2022). The participating care homes were built in the 2000s and contained about 60 beds each, a kitchen, a dining room, a living room, and a sauna, patios and balconies, as well as a single room for each resident with a private bathroom.

In 2019, the Finnish regional supervisory officials received several complaints concerning neglect in social and aged care, which made care quality a national issue with considerable media attention. The care quality and services of all four public care homes were audited by one of the authors (H. F‐S.). These care homes were invited to participate in the present study. The minimum staffing level for care personnel in units providing around‐the‐clock care for older persons in Finland was 0.60 carers per resident at the time of data collection. The purpose of the minimum staffing level is to safeguard the right of older persons to necessary care. The minimum staffing level is also essential for staff well‐being and coping at work (Ministry of Social Affairs and Health 2020).

All care home residents (*n* = 237), one of their relatives, and staff members were invited to participate in this study. Of the 237 care home residents invited, 188 participated. Additionally, 79 relatives provided responses, and staff completed proxy ratings for 145 residents. The sample included 47 resident‐relative‐staff triads. In Finnish care homes, staff are mostly enrolled nurses, while care aides and registered nurses make up a small minority. In the present study, the staff consisted of care aides (3 in each care home), enrolled nurses (36), and registered nurses (4–5), but the care aides did not participate. Each resident in the participating care homes is appointed a staff contact person who is responsible for that resident. The proxy assessment of thriving was performed as a supplement to a self‐assessment for all residents participating in this study. In the care homes, most residents were living with severe cognitive impairment.

### 4.4 Data Collection

The data collection took place in October–November 2022. This study included residents, their closest relatives (one for each resident), and the staff member who knew that resident best. The care homes were invited to participate and digital information sessions concerning the study aims and methods were arranged for managers and care staff. All participants received written information on the study. The TOPAS questionnaire was distributed to the residents by the managers and the staff. Cognitively impaired residents with CPS of 3–6 (Morris et al. 1994) were asked for consent from their respective relatives. They were not expected to perform a self‐assessment but could be provided with help from staff or their respective relatives. Residents who were under external custody, having a financial guardian, and were unable to give informed consent did not participate in this study. Most residents received help to answer the questionnaire; however, the assisting staff member was not the participating staff member who performed the assessment of thriving. A contact person appointed by the municipality sent the questionnaires to the relatives by post.

The proxy assessments were instructed to be made from a proxy‐resident perspective, that is, staff and relatives were asked to answer the survey items in the way they thought the residents themselves would have answered. All residents had either Finnish or Swedish as their native language, and all participants had the opportunity to use their native language throughout this study. The data collected from the residents included their demographic characteristics (age, gender, cohabiting status, native language, and length of stay). The participants were anonymous for the researchers, and the data were coded.

### 4.5 Data Analysis

Descriptive statistics were used to describe the participant demographic characteristics and the central interRAI scale factors. Linear mixed‐effects models were applied to test equality of the TOPAS group means between the residents, the relatives, and staff. For each TOPAS item, Cohen's weighted kappa coefficients were calculated using quadratic weights for pairwise comparisons of the interrater agreement levels of thriving for residents vs. staff, residents vs. relatives, and relatives vs. staff. Given the ordinal nature of the Likert‐scale ratings, we used quadratically weighted kappa rather than unweighted kappa. This choice allows the agreement statistic to account for the extent of disagreement, treating adjacent‐category discrepancies as less serious than disagreements spanning several response categories. To obtain valid inference under clustering by residential home, 95% confidence intervals were computed using a cluster‐robust bootstrap with 2000 replications. All statistical analyses were performed using the STATA statistical program package, version 18.

### 4.6 Ethical Considerations

The present study followed the ethical requirements of the Finnish National Board on Research Integrity (TENK 2023). In September 2022, the study was approved by the Åbo Akademi University Research Ethics Board (09/15/2022). Research permission was also obtained from the municipality that appointed a contact person for the data collection (2512/2022). Signed informed consent to participate was obtained from the residents and the relatives who also received a cover letter giving them information about consent and a notice that they could cancel their participation at any time and that cancellation of participation would not affect the residents' care. All participants, or relatives in case of severe cognitive impairment, gave informed consent.

## 5 Results

### 5.1 Characteristics of the Sample

The mean age was 86 years for residents, 65 years for relatives, and 45 years for staff. The participants were mostly female, 77%, 70%, and 97%, respectively. The mean length of stay was 29 months. Of the residents, 85% had ADL‐H > 1, 21% CHESS > 2, 84% CPS > 2, 27% DRS > 2, 29% Pain > 1, and 51% SES > 1. The participant characteristics are summarized in Table 1.

**TABLE 1:** Characteristics of the participants.

	Resident (*n* = 188)	Relative (*n* = 79)	Staff (*n* = 145)
Age (mean (SD))	85.53 (8.03)	64.52 (11.02)	44.60 (12.90)
Gender			
Female (%)	77	70	97
Male (%)	23	30	3
**interRAI**			
ADL‐H > 1	84.9		
CHESS > 2	20.5		
CPS > 2	83.6		
DRS > 2	27.4		
Pain > 1	28.8		
SES > 1	51.4		

*Note:* ADL‐H Scale 0–6, 0 where 0 represents completely independent and 6 completely dependent on daily assistance. CHESS Scale 0–5, where 0 represents extremely stable and 5 extremely unstable health. CPS Scale 0–6, where 0 represents normal cognition and 6 extremely severe cognitive impairment. DRS Scale 0–14, where 0–2 represent no depression and 3–14 probable clinical depression. Pain Scale 0–3, where 0 represents no pain and 3 extreme pain. SES Scale 0–6, where 0 represents minimal and 6 maximal social engagement (interRAI 2023).

### 5.2 Mean Ratings for the TOPAS Question Items

As shown in Table 2, residents gave the highest item‐level mean score (5.5) for Q5 ‘I regard staff as being kind’ and the lowest item‐level mean score (3.6) for Q11 ‘I have something interesting to do during the day’. The overall item‐level mean score for residents was 4.5. They used the full range of Likert‐type scale responses (1–6) on all items except Q5 ‘I regard staff as being kind’ and Q7 ‘I find that staff are competent’, where only ratings between 3 and 6 were given.

**TABLE 2:** Mean ratings and significance testing for each TOPAS item.

	Short‐form TOPAS items	Resident			Relative			Staff			*p*
		Mean	Min	Max	Mean	Min	Max	Mean	Min	Max	
1	I experience my current housing to be the best possible place to live in	4.8	1	6	5.1	2	6	4.9	1	6	0.109
2	I experience my current housing to be my home even if it is different from my previous home	4.4	1	6	4.3	1	6	4.5	1	6	0.246
3	I try to see the positive sides of being in this housing	5.0	1	6	4.9	3	6	4.7	1	6	0.252
4	I receive help that is adjusted to my needs	5.2	1	6	5.1	2	6	5.4	2	6	0.077
5	I regard staff as being kind	5.5	3	6	5.3	3	6	5.3	2	6	0.037
6	I find that staff do their best for us residents	5.3	1	6	5.1	2	6	5.3	2	6	0.111
7	I find that staff are competent	5.4	3	6	5.1	3	6	5.3	2	6	0.037
8	I have the relationship with staff that I want	5.1	1	6	4.9	2	6	5.0	2	6	0.099
9	I have nice time together with fellow residents	4.3	1	6	3.8	1	6	4.0	1	6	0.006
10	I participate in enjoyable activities	4.1	1	6	4.0	1	6	3.9	1	6	0.690
11	I have something interesting to do during the day	3.6	1	6	3.2	1	6	3.7	1	6	0.023
12	I am given possibilities to be outdoors to the extent that I wish	4.5	1	6	3.7	1	6	4.5	1	6	< 0.001
13	I am given possibilities to get out and visit places that are important for me	4.0	1	6	3.2	1	6	3.6	1	6	0.001
14	I am given possibilities to keep in contact with my relatives/friends to the extent I wish	5.1	1	6	4.9	1	6	5.2	1	6	0.084
15	I can decide how things are in my room	5.0	1	6	5.0	1	6	4.7	1	6	0.223

*Note:* The short‐form TOPAS includes 15 items and six answer options per item. Differences between raters were tested using linear mixed‐effects models with rater as a fixed effect and subject as a random effect. *p*‐values refer to tests of equality across the three raters.

Relatives gave the highest item‐level mean score (5.3) for Q5 ‘I regard staff as being kind’ and the lowest item‐level mean score (3.2) for Q11 ‘I have something interesting to do during the day’ and Q13 ‘I am given possibilities to get out and visit places that are important for me’. The overall item‐level mean score for relatives was 4.2. They used the full range of Likert‐type scale responses for most items. They rated residents' experiences lower than the residents themselves for all items except Q1 ‘I experience my current housing to be the best possible place to live in’ and Q15 ‘I can decide how things are in my room’. The largest differences between residents and relatives were observed for Q9 ‘I have nice time together with fellow residents’ (residents 4.3—relatives 3.8 = 0.5), Q12 ‘I am given possibilities to be outdoors to the extent that I wish’ (residents 4.5—relatives 3.7 = 0.8), and Q13 ‘I am given possibilities to get out and visit places that are important for me’ (residents 4.0—relatives 3.2 = 0.8).

Staff gave the highest item‐level mean score (5.4) for Q4 ‘I receive help that is adjusted to my needs’ and the lowest item‐level mean score (3.6) for Q13 ‘I am given possibilities to get out and visit places that are important for me’. The overall item‐level mean score for staff was 4.3. They used the full range of Likert‐type scale responses for most items. The differences between resident and staff item‐level ratings were quite small, ranging between 0 and 0.4. The largest differences between relatives and staff were observed for Q11 ‘I have something interesting to do during the day’ (relatives 3.2—staff 3.7 = −0.5) and Q12 ‘I am given possibilities to be outdoors to the extent that I wish’ (relatives 3.7—staff 4.5 = −0.8), both negative, illustrating the overall trend of staff giving higher ratings compared to relatives.

The linear mixed‐effects model test showed statistically significant differences among the three item‐level means (Table 2) for items Q9 ‘I have nice time together with fellow residents’, Q12 ‘I am given possibilities to be outdoors to the extent that I wish’, and Q13 ‘I am given possibilities to get out and visit places that are important for me’. Relatives gave lower ratings for almost all items compared to residents and staff.

### 5.3 Interrater Agreement Levels of Thriving as Assessed by Residents, Relatives, and Staff

Cohen's weighted kappa coefficients for the 15 TOPAS items were between 0.22 and 0.64 for resident vs. staff assessments, between 0.00 and 0.50 for resident vs. relative assessments, and between 0.04 and 0.40 for relative vs. staff assessments (Table 3).

**TABLE 3:** Cohen's weighted kappa for pairwise interrater agreement comparisons.

TOPAS items	Resident vs staff: *κ*	Resident vs relative: *κ*	Relative vs staff: *κ*
1. I experience my current housing to be the best possible place to live in	0.417 [0.264, 0.570]	0.211 [−0.069, 0.491]	0.167 [−0.041, 0.375]
2. I experience my current housing to be my home even if it is different from my previous home	0.423 [0.262, 0.584]	0.275 [0.146, 0.404]	0.260 [−0.036, 0.556]
3. I try to see the positive sides of being in this housing	0.308 [0.096, 0.520]	0.442 [0.156, 0.728]	0.263 [0.106, 0.420]
4. I receive help that is adjusted to my needs	0.259 [0.108, 0.410]	0.204 [−0.239, 0.647]	0.139 [−0.155, 0.433]
5. I regard staff as being kind	0.216 [0.022, 0.410]	0.313 [0.154, 0.472]	0.315 [0.097, 0.533]
6. I find that staff do their best for us residents	0.263 [0.122, 0.404]	0.176 [0.107, 0.245]	0.075 [−0.043, 0.193]
7. I find that staff are competent	0.387 [0.334, 0.440]	0.307 [0.166, 0.448]	0.090 [−0.032, 0.212]
8. I have the relationship with staff that I want	0.227 [0.170, 0.284]	0.497 [0.170, 0.824]	0.040 [−0.211, 0.291]
9. I have nice time together with fellow residents	0.637 [0.433, 0.841]	0.164 [−0.005, 0.333]	0.118 [−0.037, 0.273]
10. I participate in enjoyable activities	0.528 [0.442, 0.614]	0.258 [0.085, 0.431]	0.369 [0.134, 0.604]
11. I have something interesting to do during the day	0.508 [0.267, 0.749]	0.003 [−0.107, 0.113]	0.241 [−0.077, 0.559]
12. I am given possibilities to be outdoors to the extent that I wish	0.359 [−0.045, 0.763]	0.266 [−0.001, 0.533]	0.133 [−0.081, 0.347]
13. I am given possibilities to get out and visit places that are important for me	0.408 [0.243, 0.573]	0.186 [−0.149, 0.521]	0.186 [−0.061, 0.433]
14. I am given possibilities to keep in contact with my relatives/friends to the extent I wish	0.374 [0.137, 0.611]	0.210 [0.063, 0.357]	0.308 [0.157, 0.459]
15. I can decide how things are in my room	0.524 [0.185, 0.863]	0.337 [0.021, 0.653]	0.399 [0.133, 0.665]

*Note:* The interpretation of Cohen's weighted kappa coefficients was guided by the recommendation by Landis and Koch (1977): ranging from −1 to +1, level of agreement ≤ 0 poor, 0.01–0.20 slight, 0.21–0.40 fair, 0.41–0.60 moderate, 0.61–0.80 substantial, and 0.81–1.00 almost perfect. For each TOPAS item, Cohen's kappa was estimated for the three rater pairs using Stata's *kap* command (STATA 18) using quadratic weights. To obtain valid inference under clustering by care home, 95% confidence intervals were computed using a cluster‐robust bootstrap with 2000 replications.

#### 5.3.1 Interrater Agreement Levels for Thriving: Residents vs. Staff

The highest agreement (0.64) between residents and staff was obtained for Q9 ‘I have nice time together with fellow residents’ and the lowest agreement (0.22) for Q5 ‘I regard staff as being kind’ (Table 3). The agreement was at the fair level (0.22–0.39) for Q3, Q4, Q5, Q6, Q7, Q8, Q12, and Q14, at the moderate level (0.41–0.53) for Q1, Q2, Q10, Q11, Q13, and Q15, and at the substantial level (0.64) for Q9.

#### 5.3.2 Interrater Agreement Levels for Thriving: Residents vs. Relatives

The highest agreement (0.50) between residents and relatives was obtained for Q8 ‘I have the relationship with staff that I want’ and the lowest agreement (0) for Q11 ‘I have something interesting to do during the day’ (Table 3). The agreement was at the slight level (0.16–0.20) for Q4, Q6, Q9, and Q13, at the fair level (0.21–0.34) for Q1, Q2, Q5, Q7, Q10, Q12, Q14, and Q15, and at the moderate level (0.44–0.50) for Q3 and Q8.

#### 5.3.3 Interrater Agreement Levels for Thriving: Relatives vs. Staff

The highest agreement (0.40) between relatives and staff was obtained for Q15 ‘I can decide how things are in my room’ and the lowest agreement (0.04) for Q8 ‘I have the relationship with staff that I want’ (Table 3). The agreement was at the slight level (0.04–0.19) for Q1, Q4, Q6, Q7, Q8, Q9, Q12, and Q13, and at the fair level (0.24–0.40) for Q2, Q3, Q5, Q10, Q11, Q14, and Q15.

Most items showed statistically significant kappa values between residents and staff, while several confidence intervals (CI) including 0 were observed for residents vs. relatives and relatives vs. staff. Part of this variation may be explained by differences in group size, as smaller groups contribute to greater uncertainty and wider CIs, affecting both observed agreement and statistical significance. The results suggest that the groups have different perceptions, which is expected for subjective outcomes and should therefore be interpreted with caution.

## 6 Discussion

The aim of this study was to explore the interrater agreement concerning assessments of thriving among residents, relatives, and staff. The kappa coefficients for each of the 15 items ranged between fair and substantial (0.22–0.64) for resident vs. staff assessments, between poor and moderate (0.00–0.50) for resident vs. relative assessments, and between slight and fair (0.04–0.40) for relative vs. staff assessments. The highest (‘almost perfect’) levels of interrater agreement (0.81–1.00) were not observed in this study.

### 6.1 Interrater Agreement Between Residents and Staff

Items concerning social life and activities (Q9, Q10, Q11) for resident vs. staff comparisons showed the highest overall agreement (0.51–0.64), together with Q15 that also showed agreement at a similar level (0.52). The mean scores for Q9–Q11 were rather low both for residents and staff. The high agreements observed for Q9–Q11 suggest that staff were aware that social life and activities did not satisfy the needs of the residents well. The high mean and agreement values for Q15, which concerned residents' ability to decide how things are placed in their room, may indicate that staff showed respect for the residents' self‐determination and privacy.

The lowest resident vs. staff agreements (0.22–0.26) were observed for Q4, Q5, Q6, and Q8, that is, items concerning the interactions between residents and staff. When staff assess their own work there is a risk of positive rating bias. Another factor possibly leading to positive bias was the COVID‐19 pandemic during the data collection. As shown by Paananen et al. (2021), many residents were lonelier during the pandemic, and staff may have experienced their role for residents as more significant. Some units had visiting restrictions during the data collection, and there were certain differences between the care homes in the response activity. Some residents and relatives may have been unwilling to present negative assessments in relation to staff even in anonymous questionnaires, possibly feeling concerned about consequences for care. Residents have a wide range of wishes and needs, and it is important to schedule enough time to talk about these wishes and needs. The residents and staff may perceive the concept of thriving in different ways (Baxter, Sandman, et al. 2021; Sullivan and Willis 2018).

### 6.2 Interrater Agreement Between Residents and Relatives

Resident vs. relative agreements were particularly low for five items (0.00–0.20): Q4, Q6, Q9, Q11, and Q13, that is, items concerning how residents receive help and have opportunities for social activities. All agreement values were below 0.40 with the exceptions of Q3 (0.44) and Q8 (0.50). These low agreement values indicate that relatives did not know how residents experienced their daily life in the care homes. This important observation would have remained unnoticed if only the mean item‐level TOPAS ratings were considered. As an example, for Q4 ‘I receive help that is adjusted to my needs’, the item‐level means were 5.2 (residents) and 5.1 (relatives), that is, almost identical, but the agreement was only 0.20. The low agreement value was partly explained by dispersion between the responses. We did not register how often relatives visited residents, but low visit frequencies could be an explanation for the low agreement levels. Visiting restrictions due to the COVID‐19 pandemic may have reduced visit frequencies. The relatives' own concerns may also have been reflected in the results. From the relative perspective, the resident's life in the care home may appear to be of lower quality compared to their previous life, especially for residents experiencing cognitive impairment, which may be anxiety‐inducing for relatives (Robertson et al. 2020).

### 6.3 Interrater Agreement Between Relatives and Staff

The interrater agreement values were generally even lower for relative vs. staff comparisons than for resident vs. relative comparisons. For about half of the TOPAS items, the relative vs. staff agreement was fair and for the rest it was slight. The highest agreement was observed for items concerning whether residents assessed staff to be kind (Q5), participation in activities (Q10), and the possibility to decide how things are in the resident's room (Q15). Relatives may understand thriving in different ways and take into consideration the residents' life before moving to the care home. Opportunities should be offered for relatives to discuss thriving with staff.

The COVID‐19 pandemic illustrated the negative impact of social isolation in aged care and the need for competent nursing leadership to promote social engagement and contacts (Woods et al. 2022). During the data collection for the present study, the COVID‐19 pandemic limited possibilities for relatives to participate in resident care and to get acquainted with their everyday life in the care home. Due to privacy protection issues, the results were not compared between the care homes.

Our results suggest that proxies, that is, relatives and staff, assessed residents' thriving to be lower overall than the residents themselves. Römhild et al. (2018) also found that QoL self‐ratings of those with severe cognitive impairment appeared higher than the ratings obtained from staff and informal caregiver proxies. In general, the individual tends to identify less impairment than their proxies, with large differences observed in cases where proxies experience caregiver stress and individuals live with cognitive impairment (Neumann et al. 2000). In the present study, the proxy agreements differed systematically between relatives and staff, with staff showing higher agreement with residents compared to relatives. It is not uncommon for relatives to criticize the activity levels or the thriving of residents living in care homes, and staff may not always agree. In a study by Robertson et al. (2020), the relatives' fears and sense of loss influenced their assessment of QoL. Some relatives felt that it was not possible to have a good QoL in a care home.

Proxies may find it hard to answer questions concerning subjective and emotional issues, and their own expectations, needs, and impairments also can bias their assessments. The ratings of proxy respondents may show systematic differences that reflect their relationship with the resident (Neumann et al. 2000). The backgrounds of proxy respondents also may affect their ratings (Robertson et al. 2017). How proxy assessments should be used for those residents who cannot be interviewed continues to be debated (Kane et al. 2005). Robertson et al.'s (2020) study asked relatives to compare the resident's current life to a time before the cognitive impairment emerged, finding that relatives often focused on what had been lost through cognitive impairment and subsequent institutionalization. According to that study, staff use their professional understanding of impaired cognitive function and are more likely to pay attention to disease progression.

Focusing only on care routines and practical tasks, as opposed to resident preferences and well‐being, can impair thriving among both residents and staff (Vassbø et al. 2019). Relatives' satisfaction with the quality of care can be improved by providing a person‐centred environment that is secure and hospitable (Lood et al. 2020). Studies on thriving in care homes are scarce at resident, staff, and organization levels (Baxter, Corneliusson, et al. 2021). This is true also for the role of relatives. Staff should recognize the differences between resident and proxy perspectives to make accurate assessments to guide care (Howland et al. 2017).

Low agreement and wide CIs are common in studies involving individuals with dementia and proxy ratings, reflecting inherent methodological challenges (Robertson et al. 2017). The items assess residents' perceptions of thriving and daily life, which are inherently subjective. Proxy ratings introduce additional variability, as staff and relatives may interpret experiences differently, and cognitive impairment further limits response consistency (Robertson et al. 2017; Römhild et al. 2018; Griffiths et al. 2019). Small sample sizes, limited response variation, and unequal group sizes can widen CIs and lower the kappa values, as uniform or uneven responses provide limited information for estimating agreement. In this study, differences between groups were observed, and several CIs including 0 were noted, particularly in comparisons between residents vs. relatives and relatives vs. staff, indicating instances where agreement may be worse than chance. Collectively, these factors suggest that the findings should be interpreted with caution.

### 6.4 Strengths and Limitations

The sample size in our study was rather small, which limits the generalization of our findings. However, the cross‐sectional nature of the findings may be useful to map emerging trends and hypothesis generation for further study. We do not know how relatives experienced the quality of care overall, nor did we register how often relatives visited residents. The relatives' life situation and the reduced opportunity for care home visits by the COVID‐19 pandemic may have influenced their assessments. The response rate among relatives was 33.3% (*n* = 79). The reason for the low response rate may be due to several factors, such as limited insight into residents' daily lives, time constraints, emotional burden, and COVID‐19 outbreaks. This may introduce a risk of non‐response bias and affect representativeness and generalizability. The responses regarding thriving for residents who were assisted by staff in completing the questionnaires may have been influenced by the nature of this cooperation. Differences between self‐ and proxy‐rated QoL are common and vary depending on the proxy perspective. Discrepancies tend to be smaller when proxies adopt a proxy‐patient perspective and larger when a proxy‐proxy perspective is used. Proxies also tend to report lower QoL than individuals themselves, which may influence the results and should be considered in analysis and interpretation (Buchholz et al. 2024; Engel et al. 2024; Pickard and Knight 2005).

The TOPAS has not been validated in the Finnish context, but high internal consistency has been shown in the Swedish version. The TOPAS short form was translated into Finnish using a forward–backward translation method (Beaton et al. 2000). Given its validation in other Scandinavian contexts, core concepts such as ‘thriving’ are assumed to be conceptually equivalent. Data were collected in both Finnish and Swedish, with approximately half of the participants being Swedish‐speaking. These results should be interpreted with caution, and further psychometric validation of the Finnish version of TOPAS is needed. Understanding older persons' experiences of thriving in aged care is essential to develop and improve care quality.

### 6.5 Recommendations for Further Research

Studies based on larger datasets would be useful to further explore similarities and differences among resident self‐ and proxy‐assessments of thriving in care homes. The choice of proxy perspective is crucial for assessments of older persons with dementia who cannot express themselves, as it influences both the results and their interpretation (Pickard and Knight 2005; Engel et al. 2024). Considering both proxy‐patient and proxy‐proxy perspectives may therefore be particularly relevant in future thriving research, as thriving reflects a contextual and relational experience of well‐being in which ratings are shaped not only by the resident's situation but also by proxy perspectives and rater characteristics. The short‐form TOPAS needs to be validated in other contexts. It could be interesting to explore how the central interRAI scales affect the levels of interrater agreement in the assessment of older persons' thriving. Studies on how healthcare managers perceive thriving in aged care and how it is reflected in their decision‐making could also be useful.

## 7 Conclusion

The highest resident‐self and proxy interrater agreement observed in this study concerned comparisons between residents and staff for items related to social life and activities, whereas the lowest levels of agreement were observed for comparisons between relative vs. staff. Staff ratings appeared to be more closely aligned with resident‐self ratings than with relative ratings. These findings may indicate differences in how staff and relatives perceive residents´ everyday experiences and may highlight the potential value of communication between staff and relatives. Further research is needed to explore these differences and to determine how proxy assessments may best complement resident‐self assessments in care settings. The findings suggest that the Finnish version of TOPAS may have potential for use in symptom assessment in care home settings. However, as full psychometric validation has not been completed, these findings should be interpreted with caution. Further research is needed to establish the instrument's psychometric properties and to determine its suitability for broader use in Finnish aged care. Hence, our findings should be considered preliminary.

## 8 Implications for Nursing Practice

Resident, proxy, and staff assessments may provide complementary perspectives on residents' thriving and well‐being. Considering these perspectives together can help nursing staff identify similarities and differences in how residents' everyday lives and thriving are perceived and may be particularly valuable when residents have difficulties expressing their own experiences. The findings highlight the importance of strengthening communication between staff and relatives and providing relatives with opportunities to gain insight into and participate in residents' everyday lives in the care home. Information from different perspectives can support nursing staff, care‐home leaders, and decision‐makers in planning, implementing, and evaluating care that promotes thriving and well‐being among residents.

## Author Contributions

**Ann‐Sofie Silvennoinen:** conceptualization, investigation, funding acquisition, writing – original draft, methodology, project administration, resources, validation, data curation, writing – review and editing. **Edvard Johansson:** methodology, validation, visualization, writing – review and editing, software, formal analysis, data curation, supervision. **Rebecca Baxter:** writing – review and editing, supervision, resources. **Harriet Finne‐Soveri:** supervision, writing – review and editing. **Ådel Bergland:** writing – review and editing, supervision, resources. **David Edvardsson:** writing – review and editing, supervision, resources. **Lisbeth Fagerström:** writing – review and editing, supervision, conceptualization.

## Funding

This research received a grant from the Marianne Armfelt Foundation. The funding bodies had no role in the design of the study, data collection, analysis, interpretation of data, or writing the manuscript.

## Ethics Statement

This study was approved by the Åbo Akademi University Research Ethics Board (09/15/2022). Research permission was also obtained from the municipality (2512/2022).

## Consent

Participants were given written information about the study and consent was obtained. Data confidentially and participant anonymity are maintained.

## Conflicts of Interest

The authors declare no conflicts of interest.

## Supporting information


**Appendix S1:** STROBE checklist.

## Data Availability

To maintain the confidentiality of the participants' information, the data are not publicly available.
